# Incidence of pediatric invasive pneumococcal disease in the Island of Majorca (2008-2010), an area with non-universal vaccination, and estimations of serotype & children population coverage by available conjugate vaccines

**DOI:** 10.1186/1471-2334-13-503

**Published:** 2013-10-29

**Authors:** Juan Picazo, Joaquin Dueñas, Antonio Ramirez, Andres-Ricardo Perez, Emma Padilla, Susana Herrero, Carmen Gallegos, Esther Culebras, Cesar Balseiro, Cristina Mendez

**Affiliations:** 1 Microbiology Deparment, Hospital Clínico San Carlos, c/ Martín Lagos s/n, 28040 Madrid, Spain; 2 Pediatric Department, Hospital Son Dureta, c/ Andrea Doria 55, 07014 Palma de Mallorca, Spain; 3 Microbiology Department, Hospital Son Dureta, c/ Andrea Doria 55, 07014 Palma de Mallorca, Spain; 4 Pediatric Department, Hospital de Manacor, Ctra. Manacor- Alcúdia km. 1, 07500 Manacor, Mallorca, Spain; 5 Microbiology Department, Hospital de Manacor, Ctra. Manacor- Alcúdia km. 1, 07500 Manacor, Mallorca, Spain; 6 Pediatric Department, Hospital Son Llatzer, Ctra. Manacor km. 4, 07198 Palma de Mallorca, Spain; 7 Microbiology Department, Hospital Son Llatzer, Ctra. Manacor km. 4, 07198 Palma de Mallorca, Spain; 8 Medical Department, Pfizer SA, Avda. Europa 20B, 28108 Alcobendas, Madrid, Spain

**Keywords:** Majorca, PCV7, PCV10, PCV13, Invasive pneumococcal disease, Incidence rate

## Abstract

**Background:**

The World Health Organization reported in 2007 that inclusion of PCV7 in national immunization programs should be seen as a priority, also encouraging countries to conduct appropriate surveillances for monitoring the impact of vaccination. These analyses should be conducted in specific geographical areas and should be aimed to evolution of invasive pneumococcal disease (IPD), by age groups, clinical presentation, and vaccine serotypes (and non-vaccine serotypes to detect possible replacement). This study aimed to monitor the evolution of IPD incidence in children <15 years requiring hospitalization in the Island of Majorca.

**Methods:**

A prospective clinical surveillance of all culture and/or PCR-confirmed IPD in children <15 years was performed in all hospitals in the Island of Majorca (approximately 900,000 inhabitants) from January 2008 to December 2010. Incidence rate (IR) was calculated as cases/100000 inhabitants using children population data.

**Results:**

66 IPDs were identified: 39 (59.1%) parapneumonic pneumococcal empyema (PPE), 16 (24.2%) bacteremic pneumonia (BP), 7 (10.6%) primary bacteremia, 3 (4.5%) meningitis, and 1 (1.5%) osteomyelitis. IRs in the three-year study period were: 64.22 for children 12- < 24 months, 37.21 for those 24-59 months, 22.62 for those <12 months, and 3.98 for children >59 months. By study year, IRs were 21.25 in 2008, 19.89 in 2009 and 9.80 in 2010. The reduction found in 2010 was significant and due to significant reductions in IRs of IPDs caused by serotypes included in PCV10 and PCV13. Overall, estimated serotype coverage by conjugate vaccines was 12.1% for PCV7, 37.9% for PCV10 and 65.2% for PCV13. Of the 66 hospitalized children with IPD, 20 had received at least one dose of PCV7 (13 cases with identified serotype). None of these 13 cases was caused by PCV7 serotypes, all were caused by PCV13 serotypes and only 53.8% by PCV10 serotypes.

**Conclusions:**

The results of the present study evidence the importance of expanding the number of serotypes covered by PCV, and the added value of PCV13 with respect to PCV10 and PCV7, even in an area of low prevalence of 19A as the Island of Majorca.

## Background

Pneumococcal conjugate vaccines (PCV) provide serotype-specific protection, reduced carriage of vaccine-related serotypes that are often antibiotic-resistant, in the vaccinated individual as well as in the population at large through herd immunity [1]. While the use of the 7-valent PCV (PCV7) has been very effective in reducing invasive pneumococcal disease (IPD), particularly in children under two years in the USA, the impact in Europe is less certain as vaccination programs differ in individual member states [2]. The World Health Organization reported in 2007 that inclusion of PCV7 in national immunization programs should be seen as a priority, also encouraging countries to conduct appropriate surveillances for monitoring the impact of vaccination [3]. These analyses should be conducted in specific geographical areas and should be aimed to evolution of IPD, by age groups, clinical presentation, and vaccine serotypes (and non-vaccine serotypes to detect possible replacement).

In order to improve vaccine protection, vaccination with new PCV with increased serotype coverage has been proposed. PCV7 (including serotypes 4, 6B, 9V, 14, 18C, 19F and 23F) was introduced in Spain in June 2001. In November 2009 the 10-valent PCV (PCV10) including as new serotypes (in addition to those included in PCV7) serotypes 1, 5 and 7F was licensed in our country, and in June 2010 the 13-valent PCV (PCV13) was licensed with the goal of expanding serotype coverage by including, in addition to serotypes present in PCV10, serotypes 3, 6A and 19A. The three PCV were licensed for healthy children immunization and are available in Spain only in the private market except in the Autonomous Region of Madrid that approved inclusion of PCV7 in the childhood vaccination calendar in October 2006, with a switch to PCV13 in 2010, and Galicia that included PCV13 in the vaccination calendar in 2011.

The aim of this study was to monitor the evolution of IPD incidence in children requiring hospitalization in the Island of Majorca over a 3-year period (2008-2010), with analysis by age, serotype and clinical presentation.

## Methods

A prospective, hospital-based active surveillance study on IPD was carried out in all three hospitals with pediatric department located in the island of Majorca (approximately 900,000 inhabitants), Spain, all over 3 years (January 2008 - December 2010). The study population consisted in all hospitalized children (<15 years old) with laboratory confirmed IPD by culture and/or PCR. IPD was defined as presence of *S. pneumoniae*, by culture and/or PCR, in normal sterile fluids as blood, pleural fluid and cerebrospinal fluid. Written informed consent for participation in the study was obtained from parents/guardians. The Ethical Committee of the Illes Balears approved the study (IB881/07).

Basic demographic data (age, gender, PCV7 vaccination status) and clinical presentation were recorded. Samples were sent to the clinical microbiology laboratory at each center for microbiological culture and/or PCR detection. All pneumococcal isolates were sent to a single reference laboratory (Microbiology Dpt. of the Universitary Clinic Hospital in Madrid) for serotyping by Quellung reaction. Pleural and cerebrospinal fluids not yielding positive culture were also sent to the reference laboratory for being analyzed by PCR for pneumolysin (ply) and autolysin (lyt) genes [4,5]. Pneumococci confirmed by PCR were serotyped using a real-time PCR assay [6]. Susceptibility to penicillin, cefotaxime and erythromycin was determined by microdilution following CLSI recommendations [7]. Current CLSI breakpoints [8] were considered for susceptibility interpretation.

The macrolide resistance phenotypes were determined by the double disc diffusion method with erythromycin (15 μg) and clindamycin (2 μg) discs on Mueller-Hinton agar supplemented with 5% sheep blood. The plates were incubated overnight in 5% CO_2_ atmosphere at 35°C. The erm(B) and mef(A/E) genes were detected by PCR [9], with a subsequent PCR to differentiate between mef(A) and mef(E) genes [10].

Incidence rates (IRs) were calculated as the number of cases per 100 000 inhabitants using population data on children in Majorca (for each study period, for age groups and for total number of children ≤15 years of age) from Instituto Nacional de Estadistica [11]. Vaccine doses disposition data were obtained from IMS (Intercontinental Marketing Services Iberica S.A., Madrid, Spain). Per-year the estimated population covered by the vaccine was calculated by ascertaining both the population target for vaccination (children <24 months) and the estimated number of complete vaccination schedules (primary vaccination -3 doses- and the booster dose) from the number of vaccine doses sold in Majorca. Comparisons of IRs were performed with the EPIDAT version 3.1.

## Results

The number of doses of conjugate vaccines sold in Majorca was 20228 (100% PCV7) in 2008, 19986 (100% PCV7) in 2009, and 50243 (24.8% PCV7, 41.3% PCV10 and 33.9% PCV13) in 2010. The estimated target population (<24 months) covered by vaccination was 27.7% in 2008, 26.2% in 2009 and 66.8% in 2010.

A total of 66 IPDs were identified in the study period: 39 (59.1%) parapneumonic pneumococcal empyema (PPE), 16 (24.2%) bacteremic pneumonia (BP), 7 (10.6%) primary bacteremia, 3 (4.5%) meningitis, and 1 (1.5%) osteomyelitis. Mean age was 36.8 ± 26.5 months and 51.5% were males. Mean length of hospitalization was 16.3 days, with 21.5% patients requiring ICU admission. Up to 92.4% children recovered without sequelae, 6.1% with sequelae, and mortality was 1.5% (a 23-month non-vaccinated child with PPE). Table 1 shows incidence rates of IPDs, and of PPE and BP in the total pediatric population (children <15 years) by study year. Significant (p < 0.05) lower IRs of IPDs were found in 2010 compared with previous years, with a reduction of 53.9% compared to IRs in 2008 and 50.7% compared to 2009. These reductions were due to significant (p < 0.01) reductions in IRs of IPDs caused by serotypes included in PCV10 and PCV13. Incidence of PPE also decreased in 2010 (reduction of 38.4% of IRs compared to 2008 and of 44.5% of IRs compared to 2009), but reductions did not reach statistical significance. Although reductions in IRs of BP in 2010 (reduction of 76.0% of IRs compared to 2008 and of 67.1% compared to 2009) were also non-significant, significant decreases (p < 0.05) were found in BPs by PCV10 and by PCV13 between 2008 and 2010.

**Table 1:** By study year, number and incidence rates (IR) of total IPD, total PPE (parapneumonic pneumococcal empyema) and total BP (bacteremic pneumonia), and number and IR of those caused by serotypes included in PCV7, PCV10 and PCV13

	**2008**	**2009**	**2010**	**Total**
Population ≤15 years	127041	130703	132632	390376
	n (IR)	n (IR)	n (IR)	n (IR)
IPDs	27 (21.25)	26 (19.89)	13 (9.80)^a,b^	66^c^ (16.91)
PCV7-IPDs	4 (3.15)	3 (2.30)	1 (0.75)	8 (2.05)
PCV10-IPDs	17 (13.38)	7 (5.36)	1 (0.75)^a^	25 (6.40)
PCV13-IPDs	22 (17.32)	14 (10.71)	7 (5.28)^a^	43 (11.02)
PPE	14 (11.02)	16 (12.24)	9 (6.79)	39 (9.99)
PPE by PCV7	1 (0.79)	0 (0.00)	0 (0.00)	1 (0.26)
PPE by PCV10	6 (4.72)	3 (2.30)	0 (0.00)	9 (2.31)
PPE by PCV13	11 (8.66)	7 (5.36)	4 (3.02)	22 (5.64)
BP	8 (6.30)	6 (4.59)	2 (1.51)	16 (4.10)
BP by PCV7	0 (0.00)	1 (0.77)	1 (0.75)	2 (0.51)
BP by PCV10	8 (6.30)	2 (1.53)	1 (0.75)^a^	11 (2.82)
BP by PCV13	8 (6.30)	4 (3.06)	1 (0.75)^a^	13 (3.33)

^a^p ≤ 0.05 vs. 2008.

^b^p ≤ 0.05 vs. 2009.

^c^Includes 39 PPE, 16 BP, 7 primary bacteremia, 3 meningitis and 1 osteomyelitis.

Table 2 shows by study year IRs of IPDs in the different age groups (<12 months, 12- < 24 months, 24-59 months and >59 months). In the population target for vaccination (children <24 months), IRs were 49.29 in 2008, 52.52 in 2009 and 31.90 in 2010. By age group, the highest incidence was found in the group of 12- < 24 months (IR = 64.22) followed by the group of 24-59 months (IR = 37.21) and the group of children <12 months (IR = 22.62), with an incidence of only 3.98 per 100 000 inhabitants among children >59 months in the 3-year study period. While there was a non-significant increase in IRs in 2010 in children <12 months, there was a decrease in the remaining age groups. In the group of children 12- < 24 months, the reduction in IRs in 2010 vs. 2009 (79.5% reduction) reached statistical significance.

**Table 2:** By study year, number and incidence rates by age group of total IPDs and of those caused by serotypes included in PCV7, PCV10 and PCV13

	**2008**	**2009**	**2010**	**Total**
**<12 months**				
Population	8587	9484	8450	26521
	n (IR)	n (IR)	n (IR)	n (IR)
IPD	1 (11.65)	1 (10.54)	4 (47.34)	6 (22.62)
PCV7-IPDs	0 (0.00)	0 (0.00)	0 (0.00)	0 (0.00)
PCV10-IPDs	0 (0.00)	0 (0.00)	0 (0.00)	0 (0.00)
PCV13-IPDs	0 (0.00)	0 (0.00)	2 (23.67)	2 (7.54)
**12- < 24 months**				
Population	9672	9556	10358	29586
IPD	8 (82.71)	9 (94.18)	2 (19.31)^a^	19 (64.22)
PCV7-IPDs	3 (31.02)	2 (20.93)	0 (0.00)	5 (16.90)
PCV10-IPDs	4 (41.36)	2 (20.93)	0 (0.00)	6 (20.28)
PCV13-IPDs	6 (62.03)	4 (41.86)	2 (19.31)	12 (40.46)
**24-59 months**				
Population	26984	28011	28318	83313
IPD	12 (44.47)	12 (42.84)	7 (24.72)	31 (37.21)
PCV7-IPDs	1 (3.71)	1 (3.57)	1 (3.53)	3 (3.60)
PCV10-IPDs	9 (33.35)	3 (10.71)	1 (3.53)	13 (15.60)
PCV13-IPDs	11 (40.76)	7 (24.99)	3 (10.59)	21 (25.21)
**>59 months**				
Population	81798	83652	85506	250956
IPD	6 (7.34)	4 (4.78)	0 (0.00)	10 (3.98)
PCV7-IPDs	0 (0.00)	0 (0.00)	0 (0.00)	0 (0.00)
PCV10-IPDs	4 (4.89)	2 (2.39)	0 (0.00)	6 (2.39)
PCV13-IPDs	5 (6.11)	3 (3.59)	0 (0.00)	8 (3.19)

^a^p ≤ 0.05 vs. 2009.

The responsible serotype could be identified in 48 out of the 66 cases by the method used, with 18 non-typeable pneumococci. Serotype 1 was the most frequent (n = 11) followed by serotype 3 (n = 8), 7F (n = 6), 19A (n = 5), 14 (n = 4), serotypes 12 and 19F (2 isolates each), serotypes 6A, 6B, 13, 23F, 25A, 33B (1 isolate each) and serogroup 6 (n = 4). The estimated PCV coverage was: 12.1% for PCV7, 37.9% for PCV10 and 65.2% for PCV13. Of the 66 IPDs, 36 cases were confirmed by culture and 30 only by PCR. Of the 36 isolates, 31 were recovered for susceptibility testing at the central laboratory. Of the 31 isolates, two came from non-vaccinated patients with meningitis: one serotype 23B isolated in a <12 months infant and one serotype 14 from a child aged 12- < 24 months, both isolates susceptible to penicillin and cefotaxime. The 29 remaining isolates (causing non meninigitis IPDs) belonged to serotype 1 (n = 8), 7F (n = 5), 19A (n = 5), 14 (n = 3), 19F (n = 2) and six different serotypes (one isolate each). Seven isolates were fully resistant to erythromycin: four 19A isolates (3 ermB/mefE and one ermB), two serogroup 6 (erm B) and one serotype 19F (erm B/mefA). The serotype 19F isolate showed also intermediate resistance to penicillin and one of the serogroup 6 isolates also showed intermediate resistance to penicillin and cefotaxime. In addition two serotype 14 isolates showed intermediate resistance to penicillin and cefotaxime and one serotype 13 isolate showed intermediate resistance to penicillin.

Of the identified 66 children with IPD, 20 had received at least one dose of PCV7 (2 children only one dose, 5 children two doses, 11 children three doses and 2 children four doses). The serotype causing the IPD was identified in 13 out of these 20 children. None of the cases was caused by a serotype included in PCV7. Seven cases were caused by new serotypes in PCV10 (serotype 1 in 5 cases and 7F in 2 cases) and the remaining 6 cases by serotypes exclusively included in PCV13 (serotype 3 in 4 cases, and serotypes 6A and 19A in one case each).

## Discussion

The introduction of PCV7 has resulted in a marked reduction in rates of IPD by vaccine types [1]. The magnitude of the reductions is related to the prevalence of vaccine serotypes in the pre-vaccination era and to the modest increase in rates of IPDs by non-vaccine serotypes [12,13]. The present study was carried out in an area where PCV were not included in the childhood vaccination calendar, and the estimated coverage of the target population was low in 2008 and 2009 (≈27%) but highly increased to ≈ 67% in 2010 after the launch of PCV10 and PCV13. This could be related with a significant decrease in IPDs in 2010, mainly due to the decrease in IPDs by PCV10 and PCV13 serotypes, primarily occurring in children aged 12- < 24 months. This is in accordance with data from the HERACLES study in Madrid where, 11 months after the switch from PCV7 to PCV13 in the vaccination calendar, a decline in incidence rates of IPDs was observed in children <15 years of age mainly due to reductions in the age group of 12- < 24 months and in IPDs by serotype 1 and 19A [14,15].

Previous published data from our country indicated the need for expanding the coverage provided by PCV7 since among IPD cases described in a study carried out in Catalonia in 2009, 12.2% were caused by PCV7 serotypes, 51% by PCV10 serotypes, and 71.1% by PCV13 serotypes [16]. In another study in Barcelona (2007-09), 91% IPDs were caused by non-PCV7 serotypes, with serotypes 1, 19A and 3 as the most frequent [17]. The importance of non-PCV7 serotypes was also evidenced by analyzing data from areas, as Madrid, with PCV7 in the vaccination calendar, since after universal vaccination non-PCV7 serotypes caused 95.5% IPDs [18]. In the present study, the estimated serotype coverage by the different PCV greatly increased by increasing the number of serotypes included. None of the children that had received at least one PCV7 dose had an IPD caused by PVC7 serotypes, and among vaccinated children, all cases with identified serotype were caused by serotypes included in PCV13 and only 53.8% by serotypes covered by PCV10. All these facts evidence the importance of expanding the number of serotypes covered by PCV, and the added value of PCV13 (mainly by including serotypes 3 and 19A) with respect to PCV10 and PCV7. This importance would be even higher in other areas of our country where 19A is highly prevalent [18-20]. IPDs caused by serotype 19A were not found in a previous study in 2003-4 in Majorca [21], and in our series they only represented 7.6% of all IPDs. Probably a low prevalence of serotype 19A in Majorca made this serotype non-selectable by PCV7 use.

IRs of IPDs found in 2008 and 2009 in the present study in children <24 months were similar to the one previously reported in Majorca in 2003 [21]. Although the number of IPDs described in the present series is limited, thus compromising comparisons when dividing the population by clinical presentation or age group, the study included all pediatric IPDs requiring hospitalization over three years in Majorca, the largest of the Balearic Islands located in the Western Mediterranean sea.

The reduction in IRs of IPDs shown in the present study in 2010 in the total pediatric population and in children aged 12- < 24 months will probably be greater in the next years since it has been postulated that 2-3 years of universal vaccination are necessary before disease rates stabilize and higher decreases compared to baseline are shown [1,12,13], and second-generation PCVs are increasingly being used in Majorca. In this sense, decision analytic models evaluating health and economic outcomes have shown that universal PCV13 vaccination would be a cost-effective intervention in other areas of Spain as Valencia or of Europe as Switzerland [22,23].

## Conclusions

The results of the present study evidence the importance of expanding the number of serotypes covered by PCV, and the added value of PCV13 with respect to PCV10 and PCV7, even in an area of low prevalence of 19A as the island of Majorca.

## Abbreviations

BP: Bacteremic pneumonia; IPD: Invasive pneumococcal disease; IR: Incidence rate; PCR: Polymerase chain reaction; PCV: Pneumococcal conjugate vaccine; PCV7: 7-valent pneumococcal conjugate vaccine; PCV10: 10-valent pneumococcal conjugate vaccine; PCV13: 13-valent pneumococcal conjugate vaccine; PPE: Parapneumonic pneumococcal empyema.

## Competing interests

C.B. and C.M. are employees of Pfizer SA not owing stock options of the company. J.P has received travel grants for conferences and presentations. Other authors have not conflicts of interest.

## Authors’ contributions

CB and CM have participated in the study design. All other authors have contributed to study design, acquisition of data, critical review of the manuscript and final approval of the version submitted. All authors read and approved the final manuscript.

## Pre-publication history

The pre-publication history for this paper can be accessed here:

http://www.biomedcentral.com/1471-2334/13/503/prepub
